# Diagnostic biomarker discovery from brain EEG data using LSTM, reservoir-SNN, and NeuCube methods in a pilot study comparing epilepsy and migraine

**DOI:** 10.1038/s41598-024-60996-6

**Published:** 2024-05-09

**Authors:** Samaneh Alsadat Saeedinia, Mohammad Reza Jahed-Motlagh, Abbas Tafakhori, Nikola Kirilov Kasabov

**Affiliations:** 1 Complex Systems Research Laboratory, Iran University of Science and Technology, Tehran, Iran; 2 Department of Neurology, School of Medicine, Iranian Center of Neurological Research, Tehran University of Medical Sciences, Tehran, Iran; 3 School of Engineering, Computing and Mathematical Sciences, Auckland University of Technology, Auckland, New Zealand; 4 Institute for Information and Communication Technology, Bulgarian Academy of Sciences, Sofia, Bulgaria; 5 Computer Science and Engineering Department, Dalian University, Dalian, China

**Keywords:** Spike encoding algorithm, Epilepsy, Pattern recognition, Migraine, EEG classification, Deep BiLSTM, Deep reservoir SNN, NeuCube, Biomarkers, Computational biology and bioinformatics, Classification and taxonomy, Computational neuroscience, Machine learning, Network topology

## Abstract

The study introduces a new online spike encoding algorithm for spiking neural networks (SNN) and suggests new methods for learning and identifying diagnostic biomarkers using three prominent deep learning neural network models: deep BiLSTM, reservoir SNN, and NeuCube. EEG data from datasets related to epilepsy, migraine, and healthy subjects are employed. Results reveal that BiLSTM hidden neurons capture biological significance, while reservoir SNN activities and NeuCube spiking dynamics identify EEG channels as diagnostic biomarkers. BiLSTM and reservoir SNN achieve 90 and 85% classification accuracy, while NeuCube achieves 97%, all methods pinpointing potential biomarkers like T6, F7, C4, and F8. The research bears implications for refining online EEG classification, analysis, and early brain state diagnosis, enhancing AI models with interpretability and discovery. The proposed techniques hold promise for streamlined brain-computer interfaces and clinical applications, representing a significant advancement in pattern discovery across the three most popular neural network methods for addressing a crucial problem. Further research is planned to study how early can these diagnostic biomarkers predict an onset of brain states.

## Introduction

Diagnosing brain states is crucial in clinical practice to ensure preventive healthcare. Finding a small number of diagnostic brain markers would allow for the development of wearable devices that can early detect brain states and discriminate different brain states that would be manifested similarly. For example, online discrimination between epilepsy and migraine, using a small number of mounted EEG electrodes, is still an open question addressed in this paper. A solution to the problem is offered using proposed methods for biomarker discovery for three state-of-the-art techniques, namely deep LSTM, a reservoir spiking neural network (SNN), and a brain-inspired SNN (NeuCube).

This study not only presents solutions to the discrimination problem but also conducts a comprehensive comparison of the proposed techniques, both theoretically and through case study data analysis. Furthermore, the paper introduces potential biomarkers, specific to distinguishing epilepsy from migraine, when using EEG data. Notably, amidst various deep structures such as^1–3^, that have shown promise in neurological disorders classification using MRI processing approaches, this research uniquely focuses on EEG as time series data, emphasizing mere EEG classification techniques.

Against the backdrop of the well-established training procedures and the significance of large datasets in deep learning, this study accentuates persistent challenges. Small datasets, overlapping manifestations, and the demand for efficient online or batch processing to optimize computation time emerge as critical gaps. Within this context, the research investigates the suitability of different structures, including the selection of EEG channels, for effective brain state diagnosis.

Dealing with small and unbalanced datasets in machine learning classification problems is a crucial challenge, especially when distinguishing between migraine and epilepsy using EEG data. Various techniques can be employed to mitigate the challenges posed by small datasets, such as cost-sensitive learning, synthetic data generation, and careful selection of classifiers^4^. However, justifying the use of small datasets in deep learning is essential, and it is important to consider the impact of dataset size on the quality of the mapping function in deep learning models^5^.

Migraine is a recurrent neurological disorder characterized by episodes of moderate to severe headache and potential neurological disturbances. Migraine involves dysregulation of both cortical and subcortical brain regions^6,7^.

Recurrent, uncontrolled seizures characterize epilepsy. The primary cause of seizures is abnormal and excessive activity of neurons in the brain. This activity is usually associated with the abnormal synchronous firing of neurons^8^.

Therefore, both migraine and epilepsy are neurological disorders that involve changes in the electrical activity of the brain. Although both conditions share some commonalities, they have distinct and overlapping pathways responsible for their symptoms. The rationale for distinguishing between epilepsy and migraine lies in their overlapping clinical features, comorbid relationships, and the need for effective treatment decisions. While both epilepsy and migraine are episodic brain disorders with some similar characteristics, including headache pain and a pre-attack phase called aura, they are distinct neurological diseases with specific clinical features and underlying pathophysiological mechanisms^9^. It is essential to differentiate between the two due to their comorbid relationship, as migraine is expected in almost a quarter of patients with epilepsy, and epilepsy is anticipated in 1–2% of migraine sufferers^10^. Furthermore, some migraine patients may exhibit epileptic discharges by EEG recording, leading to potential misdiagnosis^11^. Therefore, accurate diagnosis is essential to provide appropriate and targeted treatment for each condition, considering their unique aspects, triggers, and mechanistically based therapeutic overlap^9^.

The effects of these two conditions on neural activity of the brain are significant. In epilepsy, abnormal neural activity can lead to seizures, which can be disabling and debilitating. In migraine, the changes in neural activity involve the same neurotransmitter and receptor systems as those involved in epilepsy. However, in migraine, the primary target affected is the cortex rather than the entire brain. Research has also demonstrated that in some cases, migraine is associated with altered or decreased cortical function^6^. Both epilepsy and migraine are characterized by abnormal brain electrical activity, measured by EEG^6^. Studying the impact of these brain dysfunctions on brain neural activity is critical for developing more precise and efficient treatments^7^.

EEG signals measure electrical activity in the brain and can yield various results depending on the type of disorder and its severity. A proper understanding of the effects of migraines and epilepsy on EEG classifications is vital for the reliable diagnosis and treatment of these disorders. EEG readings differ significantly between patients with epilepsy and migraines. In individuals with epilepsy, the EEG reads a high amplitude of electrical activity, usually seen in spikes or sharp waves. In these cases, the patient often displays abnormal or “epileptiform” patterns. In patients with migraine, the EEG signals often exhibit a lower amplitude of activity, which can sometimes be normal in certain cases. Additionally, there is usually a series of sharp waves or spikes in the EEG traces of migraine sufferers. Furthermore, their EEG signal trace may show slowed or irregular rhythms or a pattern that is slower than that of normal subjects^8^.

In addition to these differences in activity levels, EEG signals from patients with migraines can also reveal a deficiency of normal functions. For instance, an EEG of a migraine patient might reveal a decrease in alpha wave activity, which is a normal neural rhythm that can be found in healthy individuals^12^. Additionally, migraine recordings might illustrate a decrease in gamma waves, which are relatively high-frequency brainwaves that are associated with cognitive functioning. The classification of both types of EEG signals is further complicated when visual analysis is taken into consideration. Visual analysis assesses the patterns and waves of EEG recordings to make a diagnosis, and it may reveal different results for different patients. In patients with epilepsy, the brain waves can look abnormally shallow during the seizure-free phase, and this anomaly is called a “phasic low-amplitude rhythm shift.” On the other hand, in migraine patients, the EEG may look normal, which can make it harder to diagnose correctly^6,8^.

The impact of migraines and epilepsy on EEG classification is a complex matter that heavily relies on visual and numerical analysis of the data. Although both conditions share similar physiological responses, they require varying levels of treatment. This difference in symptoms affects how physicians diagnose and choose treatment options for each condition, making it crucial to have a thorough understanding of the impact of each disorder on EEG classifications^6,8^. To tackle this challenge, researchers are increasingly leveraging Machine Learning (ML) for crafting highly precise classification systems^10^. By leveraging ML, medical practitioners can analyze EEG data more effectively and precisely, which ultimately results in enhanced diagnoses and treatment alternatives for their patients. As such, the use of ML algorithms to classify signals in EEG recordings has become increasingly important in the early diagnosis and management of these conditions, especially when this is based on a set of discovered features/markers. Based on previous performance, three such techniques, deep BilLSTM^14^, reservoir SNN (RSNN)^15^ and a brain-inspired SNN NeuCube^16^ are proposed here as solutions to the problem and new methods for the discovery of features to discriminate EEG signals of epilepsy, migraine, and healthy subjects are developed in this paper.

Due to its capacity to handle both forward and backward information flow, BiLSTM is a sort of recurrent neural network that is frequently used for sequence modeling tasks. This makes it suitable for EEG data since it can capture long-term temporal dependencies in the signal^17^. SNNs, on the other hand, can recognize variations in the electrical activity of the brain and mirror the behavior of genuine neurons, making them well-suited to manage the variability of EEG data^14^, but neither approach has been used to discover features/biomarkers from EEG data^15^.

In a recent study, Ref^18^ presents an automated seizure classification technique using nonlinear higher-order statistics and deep neural network algorithms. The study describes a computationally fast seizure classification algorithm that achieves reliable classification accuracy for both binary classes and three classes of electroencephalogram (EEG) signals with the softmax classifier. The third-order cumulant coefficients matrix is analyzed using a stack autoencoder-based deep neural network to extract key structural details, enhancing the reproducibility and robustness of the results.

In a different investigation, a novel approach utilizing BiLSTM for classifying emotions based on EEG signals showed favorable results, demonstrating its effectiveness in emotion categorization^19^. Another study^19^ introduced a unique method incorporating a residual time–frequency wavelet analogy to analyze EEG signals in a high-dimensional space. This involved utilizing a bi-LSTM-based deep learning network, ensuring rapid and accurate classification with maximum probability. Another comprehensive review delved into the utilization of EEG signals for evaluating diverse cognitive activities, particularly excelling in emotion recognition due to their spatial resolution and suitability for this purpose^21^. Building on this foundation, a subsequent study^20^ implemented a moving window technique based on Multi-class Common Spatial Patterns to extract distinct spatial patterns from EEG data. The integration of BiLSTM into the framework was strategically designed to enhance classification outcomes. The interplay between spatial pattern and spatial resolution lies in the fact that spatial patterns reflect the distribution of neural activity, with spatial resolution determining the level of detail and accuracy in localization and differentiation, which are not independent.

Beyond emotion classification, BiLSTM has been attempted also for pattern detection of migraine and epilepsy. According to a study, a BiLSTM model may accurately identify epilepsy from EEG signals^21^. Another study employed a BiLSTM network to analyze EEG signals and forecast the occurrence of migraines. The BiLSTM network outperformed other techniques, including support vector machines (SVM) and convolutional neural networks (CNN) in terms of accuracy^22^, but BiLSTM models have not been used to discriminate epilepsy from migraine and here EEG signals are slightly different, only if considered in space and time.

BiLSTM models have difficulties when used to categorize EEG data for medical applications. These difficulties include the necessity for a significant amount of labeled data, deciphering the model’s output, and optimizing the network design and hyperparameters^23^. To overcome these challenges, researchers have developed improved BiLSTM models and have used transfer learning to reduce the amount of labeled data required for training^23^. In our paper, we have proposed a deep BiLSTM.

Another approach to EEG classification is the use of spiking neural networks (SNNs) and reservoir SNNs (RSNNs) in particular. SNNs are effective in identifying changes in the electrical activity of the brain and can accurately detect seizures and migraine attacks^24^. However, challenges remain in training RSNNs on a large number of labeled samples to accurately classify the data, as EEG data sets are often small or nonexistent for some disorders^25^. RSNNs also require a large amount of computing power to accurately train the neural networks and identify patterns^26^. Furthermore, EEG data is often noisy and difficult to interpret, making it challenging for RSNNs to make accurate predictions^27^.

While both BiLSTM and RSNN models have been used in the classification of EEG data, they each face unique challenges. For example, both BiLSTMs and RSNNs face challenges in accurately classifying EEG data associated with migraine and epilepsy. In the interictal phase, both conditions can present overlapping features in the EEG of healthy subjects and migraine patients, leading to an increased risk of misclassification^28^. Furthermore, most studies in the field of EEG classification using deep learning methods have focused on examining and diagnosing migraine and epilepsy separately from healthy people, with fewer studies investigating both groups simultaneously^29^. Finally, most studies in this field take advantage of time–frequency features as their input data, which can significantly increase the computational load in these two types of networks^30,31^.

While both the BiLSTM and the RSNN can learn effectively temporal data, the brain-inspired SNN NeuCube can perform deep learning of both spatial and temporal elements from spatiotemporal data, which makes it superior for spatiotemporal brain data, such as EEG and fMRI^16,32,33^. NeuCube is based on an RSNN that is structured according to a brain template, such as Talairach or MNI^33^, thus allowing for the 3D SNN to capture spatio-temporal patterns. It uses the STDP local learning rule to capture global spatio-temporal patterns from the spatio-temporal data^16^.

Despite advancements in studying EEG data, elucidating identified patterns and defining effective biomarkers remains a formidable task. To tackle this challenge, our investigation delved into the potential of concealed neurons in the diagnosis of neurological disorders. We scrutinized the operations of hidden neurons in three profound deep-learning networks—namely, deep BiLSTM, extended Recurrent Spiking Neural Network (RSNN), and NeuCube. Our endeavor introduces an innovative adaptive spike detection algorithm for SNNs, leveraging online spiking thresholds coupled with combined Reinforcement Spiking Neural Network with STDP Learning (ReSuMe-STDP) learning. This algorithm adapts to statistical attributes, heightening pattern discernibility and analysis quality by updating encoding thresholds. To underscore the significance of adaptive threshold spike detection in ambient noise, we also present techniques for extracting meaningful patterns and biomarkers from each deep network model. Furthermore, our paper addresses hurdles in accessing and recording voluminous datasets for Electroencephalogram (EEG)-based decision-making in the context of epilepsy treatment, by ascertaining the efficacy of the classifier model. We compare the suitability of diverse architectures, emphasizing rational connections in deep learning—specifically, juxtaposing Deep Artificial Neural Networks (ANNs), Long Short-Term Memory (LSTM), and SNNs for temporal series analysis. Our investigation proffers potential biomarkers to differentiate epilepsy from migraine using EEG data, underlining persistent challenges in deep learning, such as limited datasets and overlapping manifestations. The novelty of our work lies also in the proposed online spike encoding approach for SNNs, amplifying real-time performance via mean and standard deviation computations, online normalization, and adaptive threshold adjustments. Moreover, we introduce a novel deep SNN classifier structure endowed with expeditious processing capabilities, providing invaluable insights for effectively distinguishing epilepsy from migraine.

The paper’s structure is organized as follows: “Materials and Methods” commences by detailing a neuronal model, followed by the introduction of the novel ReSuMe-STDP reinforcement learning for an RSNN. It further presents the innovative Online Spike Encoding Algorithm and elucidates the architecture of the proposed deep BiLSTM classifier. The section then delves into the methods employed for pattern recognition and diagnostic marker discovery utilizing deep BiLSTM, proposed ReSuME-STDP-based RSNN structure (RSM-SNN), and NeuCube models, culminating in a comparative analysis focused on the epilepsy versus migraine case study. Data specifications are expounded upon subsequently. Moving to “Results”, the paper examines the proposed method’s efficacy in discovering patterns within BiLSTM hidden neurons, observes firing patterns within the RSM-SNN, and uncovers diagnostic biomarkers using NeuCube models. Additionally, encoding performance is rigorously analyzed. The paper draws to a close with a comprehensive “Conclusion”, followed by an “Acknowledgment” section and a list of references.

## Materials and methods

This section consists of three main subsections. The first subsection proposes a new spike encoding method combined with a partially observed ReSuMe STDP recurrent SNN structure. This approach creates a new online architecture for classifying EEG signals to distinguish between Epilepsy, Migraine, and healthy subjects. The second part introduces the BiLSTM structure, which was used as a basis for comparison in our study. The third part describes the NeuCube structure as the third architecture to classify EEG signals and analyze their performances and hidden neuron activities.

All methods were carried out under relevant guidelines and regulations. The Iranian Center of Neurological Research approved the experimental protocols. Informed consent was obtained from all subjects and their legal guardian(s) before collecting the data. This data was used under ethical guidelines and regulations on issues related to informed consent, confidentiality, and other ethical considerations.

### Proposed deep RSM_SNN structure

**Figure 1:**
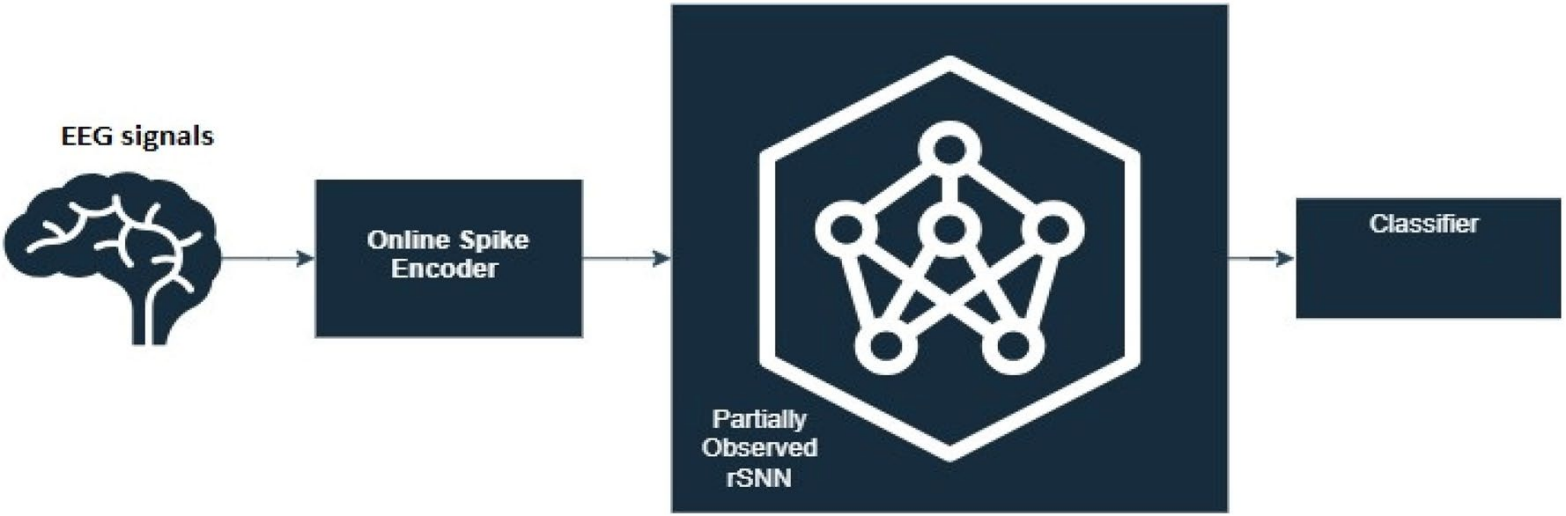
Main structure of proposed online RSM-SNN.

Figure 1 illustrates the proposed method’s primary structure and demonstrates the modules’ interrelationships.

The proposed structure, as shown in Fig. 1, involves several steps. The first step is to receive raw online EEG data. The Online Spike encoder then adaptively encodes the data and inputs the spike trains into the Partially Observed reservoir SNN (rSNN). The rSNN is a variant of the SNN that capitalizes on the notion of a “reservoir” comprising neurons that are recurrently interconnected to handle temporal information. SNNs draw inspiration from the spiking neurons in the biological brain and are renowned for their event-triggered computation, thus rendering them apt for the modeling and processing of spatiotemporal data, such as EEG signals^13^. The rSNNs are utilized in the analysis of EEG due to their capability to handle unpredictability and stochastic characteristics present in brain signals. The intrinsic parallelism and abundant dynamics of SNNs render them well-suited for modeling the intricate and dynamic nature of EEG data. Furthermore, the stochastic connectivity within the reservoir enables efficient processing of stochastic patterns in EEG signals. The capability of SNNs to capture temporal dynamics and exploit the stochasticity inherent in neural activities corresponds with the unpredictable nature of EEG signals, facilitating effective analysis and deciphering of brain activity^32^.

The proposed output module classifies the pattern of output spikes using a simple average firing pattern isolation. This classifier organizes the EEG signals into three groups—Epilepsy, Migraine, and healthy.

The network structure proposed and utilized in this paper is inspired by the three-dimensional structure of MRI-SNN, presented in Ref^34^. This structure has two groups of neurons, observed and hidden. The observed neurons are trained using the supervised learning approach, while the hidden neurons use the STDP learning law to update the synaptic weights. ReSuMe learning is utilized in the observed neurons. It is worth noting that the proposed structure in this article differs from the MRI-SNNr structure^34^ in the use of the ReSuMe learning rule instead of the presented learning rule in Ref^34^. Additionally, the coding process and arrangement are also different.

**Figure 2:**
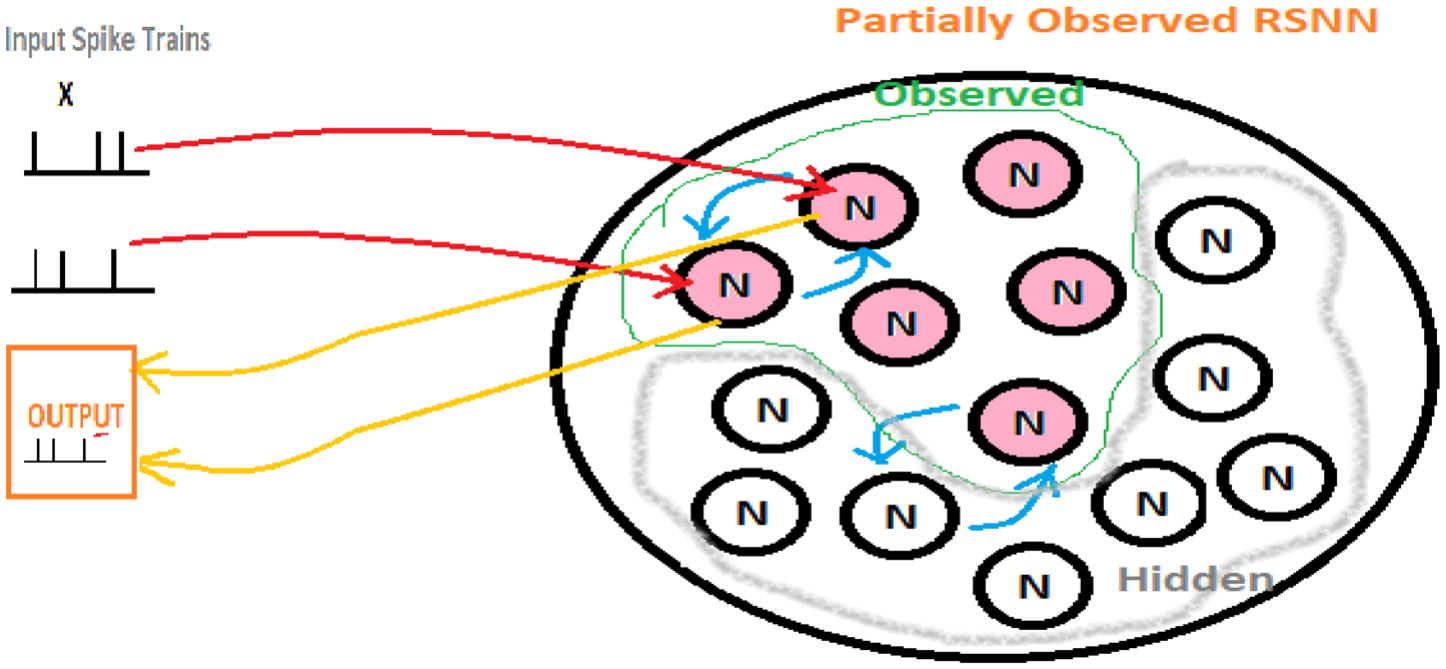
A schema of the proposed rSNN.

The network used for data classification in this study is arranged in two dimensions, as it is trained based on temporal data, and spatial data is not considered. Therefore, the preprocessing of spatial data has been eliminated, and the ability to analyze raw data by the proposed online spike encoder makes the proposed structure a deep architecture, which the output module filtering layer can enhance as a classifier or even a regulator. Figure 2 indicates the structure of the proposed rSNN using the ResuMe-STDP learning law. This approach is similar to the input data to BiLSTM, which allows for better comparison.

The proposed rSNN consists of fully connected Izhikevich neuron models. This neuron model is simple and biologically plausible, and it can replicate most known firing patterns. The equations of the neuron are given below according to Ref^19^.

$$ \dot{V} = 0.04V^{2} + 5V + 140 - u + I_{noise} + I $$

$$ \dot{u} = a\left( {bV - u} \right) $$

1 $$ {\text{if}}\; V \ge 30\;{\text{ then}}\left\{ {\begin{array}{*{20}l} {V = c} \hfill \\ {u = u + d} \hfill \\ \end{array} } \right. $$

where v is the membrane potential, I and I_noise are the synaptic and spontaneous background noise currents, respectively, and u is the slow recovery variable. For excitatory neurons (EXs), the rules of the parameters a (time scale of recovery) and b (sensitivity of recovery) are considered to be between 0.02 and 0.2, respectively. For EXs parameters, c ranges from − 65 to − 50 mv, and d (recovery after spike) ranges from 6 to 8. Inhibitory neurons (INs) have fixed parameters: c at − 65 mV and d at 2^35^. The Izhikevich neural network comprises 70% EXs and 30% INs.

#### A proposed online spike encoding algorithm (OSE)

Encoding input data is a crucial step in the supervised training of spiking neural networks (SNN). It can significantly impact network performance, particularly in online progress in areas such as robotics and brain-computer interfaces^36^. Several spike encoding algorithms are available to address this issue, including threshold-based encoding, Bens Spikes Algorithm, and others^32,33^. Ref^37^ presents a method to evaluate spike encoding algorithms based on the type of input time series.

This paper introduces a new online spike detection algorithm that can be considered a self-tuning threshold spike detection algorithm. The fundamental concept of the proposed algorithm is based on the input data rate. The paper describes the recurrent spike detector in detail. This algorithm adaptively updates the mean rate of input signals and its variation, and then based on the calculated variation rate and updating mean of the signal in each epoch, it updates the standard variation and expected value of the signal, and adapts the threshold of the encoding algorithm recursively based only on the last recorded data to enhance reliability and speed up the process of encoding. This makes rSNN act as a deep learning processor and eliminates noise mean effects by calculating the average and considering a threshold higher than the mean as a criterion for encoding. The algorithm’s novelty lies in its adaptive spike encoding approach for SNNs, amplifying real-time performance via mean and standard deviation computations, online normalization, and adaptive threshold adjustments.

**Table:** 

OSE algorithm		
(1)	Initializing: $$\begin{gathered} \begin{array}{*{20}c} {} & {m_{in} (0) = 0} & {std_{in} (0) = 1} \\ \end{array} \begin{array}{*{20}c} , & {} \\ \end{array} \hfill \\ \begin{array}{*{20}c} {} & {m_{v} (0) = 0} & {std_{v} (0) = 1} \\ \end{array} \hfill \\ \end{gathered}$$	
(2)	Calculating the rate of variation of input data: $V_{{x_{in} }} (k) = \frac{{X_{in} (k) - X_{in} (k - 1)}}{\Delta t}$	(2)
(3)	Calculating the average variation rate from the first time step to the *k*th: $m_{in} (k) = \frac{{\left( {k - 1} \right)m_{in} (k - 1) + V_{{x_{in} }} (k)}}{k}$	(3)
(4)	Calculating the standard deviation of input data rate: $std_{in} (k) = \frac{{\left( {k - 1} \right)std_{in} (k - 1) + \left| {V_{{x_{in} }} (k) - std_{in} (k - 1)} \right|}}{k}$	(4)
(5)	Calculate normalized rate of variation: $V_{Nx} = \frac{{V_{{x_{in} }} (k) - m_{in} (k)}}{{std_{in} (k)}}$	(5)
(6)	Calculating the average of the normalized input signal: $m_{v} (k) = \frac{{\left( {k - 1} \right)m_{v} (k - 1) + V_{Nx} (k)}}{k}$	(6)
(7)	Calculating the standard deviation of the normalized input signal: $std_{v} (k) = \frac{{\left( {k - 1} \right)std_{v} (k - 1) + \left| {V_{Nx} (k) - std_{v} (k - 1)} \right|}}{k}$	(7)
(8)	Encoding input signal based on variation rates and statistics features: $$X(k) = \left\{ {\begin{array}{*{20}l} 1 \hfill & {if\;V_{Nx} (k) \ge m_{v} (k) + {{std_{v} (k)} \mathord{\left/ {\vphantom {{std_{v} (k)} 2}} \right. \kern-0pt} 2}} \hfill \\ 0 \hfill & {{\text{else}}} \hfill \\ \end{array} } \right.$$	(8)

where $X_{in} (k)$ is the input data at the *k* time step. $m_{in}$ and $std_{in}$ are respectively mean and standard deviation of the input data rate. $V_{{x_{in} }} (k)$ and $V_{Nx}$ are respectively the rate of the input and the normalized one. $m_{v} (k)$ and $std_{v} (k)$ are the mean and standard deviation of the normalized rate. $X(k)$ is the spike train input. The proposed spike encoding algorithm is suitable for online spike encoding of raw signals with noise, and consequently for deep learning. It is compared favorably with the classical BSA in a later section of this paper.

#### ReSuMe-STDP learning law

This section introduces an innovative algorithm that integrates the ReSuMe learning algorithm^38^ with the TDP algorithm. This integration aims to achieve reinforcement learning in a Spiking Neural Network (RSNN). The updating synaptic weights law in ReSuMe is described as follows:^31,33,34^

9 $$ W_{di} \left( s \right) = \left| {\begin{array}{*{20}l} {a_{di} \left( { - s} \right) = A_{ + } e^{{ + \left( {\frac{s}{{\tau_{ + } }}} \right)}} } \hfill & {s \le 0} \hfill \\ {a_{id} \left( s \right) = A_{ - } e^{{ - \left( {\frac{s}{{\tau_{ - } }}} \right)}} } \hfill & {s > 0} \hfill \\ \end{array} } \right.i \in \vartheta $$

10 $$ \dot{w}_{ij} = \left[ {S_{d} \left( t \right){ - }S_{i} \left( t \right)} \right]\left[ {a_{d} + \int\limits_{0}^{\infty } {a_{dj} \left( s \right)S_{j} \left( {t{ - }s} \right)ds} } \right]\quad i \in \vartheta \;j \in \vartheta \cup H $$

where $\dot{w}_{ij}$ $\dot{{{\text{w}}}_{{\text{ij}}}}$ $\dot{{{\text{w}}}_{{\text{ij}}}}$ is the synaptic weight rate and s is the time difference between the spike time of the ith neuron and the postsynaptic neuron j. $A_{ + } ,A_{ - } > 0$ are the maximum values of synaptic modification and $\tau_{ + } ,\tau_{ - } > 0$ represents the time constant of permissible changes in synaptic weights. $a_{d}$ is a non-correlative factor adjusting the average strength of the synaptic inputs. $S_{d} (t)$, $S_{i} (t)$ and $S_{j} (t)$ are respectively target spike trains, ith neuron spike trains, and jth neuron spike trains. In order to train the hidden neurons, the STDP rule is applied to reinforce the biological plausibility concept of the network. The synaptic weight updating law of STDP is described as follows:

11 $$ \Delta w_{ij} = \left\{ {\begin{array}{*{20}l} {A_{ + } e^{{ - \left( {\frac{s}{{\tau_{ + } }}} \right)}} } \hfill & {s \le 0} \hfill \\ 0 \hfill & {s > 0} \hfill \\ \end{array} } \right. $$

where $\Delta w_{ij}$ is the synaptic weight change per sample time.

In the upcoming section, we explore the outcomes of our data classification and scrutinize the activity patterns of neurons. This helps to understand the brain processes at operation more thoroughly and uncover the mechanisms underlying the discoveries. We can get important insights into the neural activity of both networks, BiLSTM and SNN, and their potential to deliver further knowledge about the effects of illnesses on the brain by exploring these important areas of our research.

### The proposed deep BiLSTM classifier structure

LSTM is a recurrent neural network (RNN) designed to overcome the vanishing and exploding gradient problems associated with training traditional RNNs. It is highly appropriate for consecutive tasks such as voice perception, linguistic pattern formation, and temporal series estimation due to its capability to acquire and recollect across prolonged successions. The “bi” in BiLSTM signifies its bidirectional characteristic, enabling it to handle the input sequence in both progressive and regressive orientations, which can seize a more all-encompassing context for the input information^39,40^.

The BiLSTM framework is utilized for the categorization of variability and stochastic data. The distinctive proficiencies of the BiLSTM model render it highly appropriate for this undertaking. The sequential processing capability of the BiLSTM enables it to effectively manage the temporal characteristics and innate variability present in time-series or stochastic datasets. Its bidirectional nature facilitates the comprehension of interdependencies from both preceding and subsequent data points, augmenting its capacity to retain and employ critical information for comprehending stochastic patterns. Additionally, the model’s ability to grasp long-term interdependencies and its adaptability in acquiring knowledge equip it with the proficiency to classify datasets characterized by variability and stochasticity. Furthermore, the BiLSTM’s contextual comprehension, attained by considering both preceding and subsequent contexts for each data point, furnishes a comprehensive understanding of the data’s temporal dynamics, which is pivotal for precisely categorizing stochastic and randomly evolving patterns^41^. The proposed here deep BiLSTM classifier consists of the following layers:

- BiLSTM layer: The input data is first passed through a BiLSTM layer with e.g. 50 units to capture the temporal dependencies and extract relevant features.
- Fully connected layer: The output of the BiLSTM layer is then passed through a fully connected layer to further refine the features and extract more discriminative information.
- Softmax layer: The output of the fully connected layer is then passed through a softmax layer to obtain the class probabilities.
- Cross-entropy layer: Finally, a cross-entropy layer is used as the loss function to optimize the model during training.

**Figure 3:**
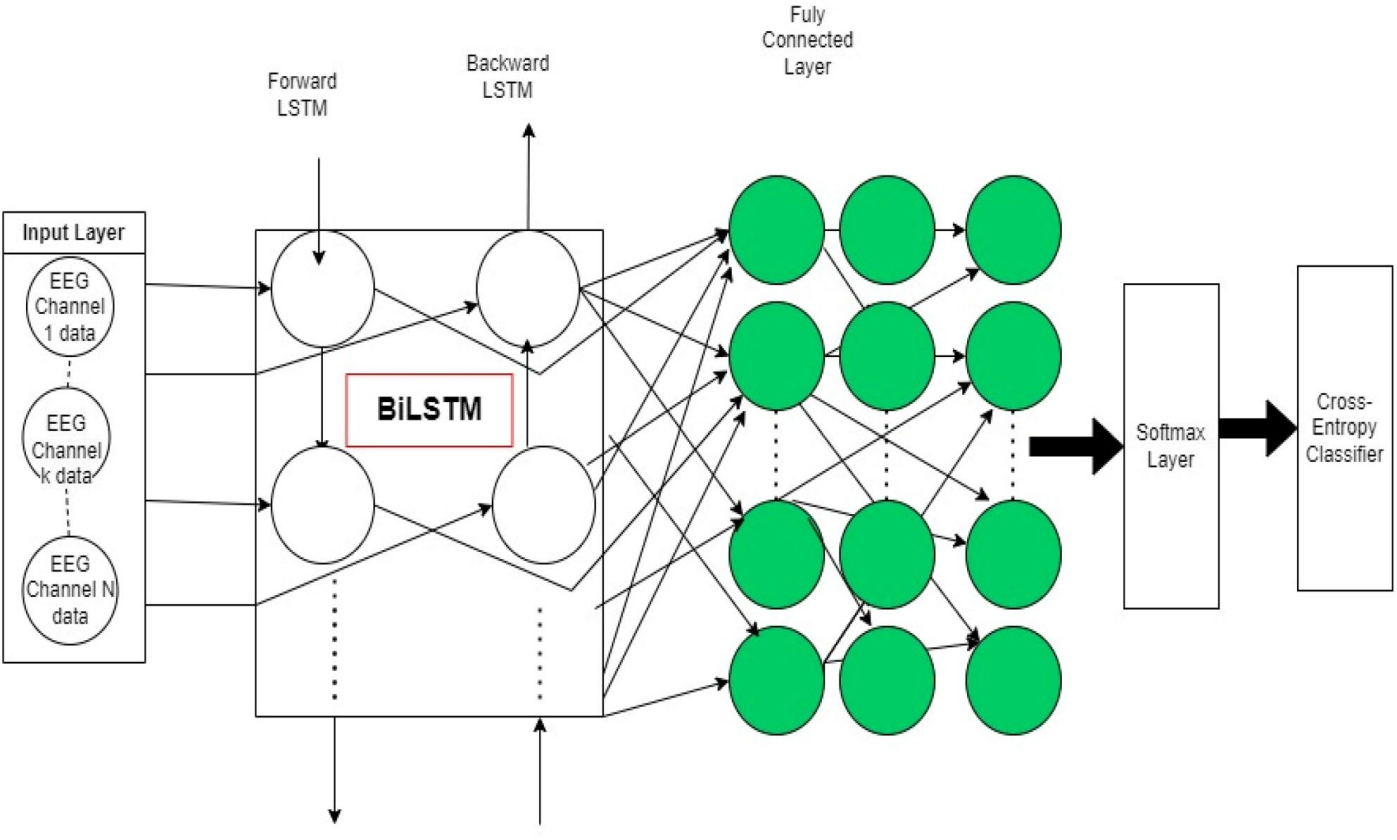
The proposed architecture of a deep BiLSTM classifier for EEG data.

First, we load the raw EEG data into the model to implement the network. Then, the data is passed through a BiLSTM layer consisting of 50 units. Next, we have three fully connected layers and a softmax layer. Finally, we use a cross-entropy layer to calculate the loss between the predicted and ground truth labels. To ensure efficient training, we set the batch size to 25. Figure 3 indicates the overall structure of the utilized BiLSTM classifier.

### The NeuCube brain-inspired SNN architecture

NeuCube is a specialized spiking neural network architecture designed to model and analyze complex spatiotemporal data, especially from brain and cognitive systems^16^. It uses a 3D SNN model inspired by the structural organization of the human brain, which helps it capture deep spatio-temporal dynamics. This design makes it particularly effective for tasks such as classifying EEG signals and diagnosing brain states^32,33^. The NeuCube is specifically employed for EEG analysis due to its capability to handle randomness and stochastic features in the brain’s electrical signals. The self-programmable and learning abilities of NeuCube make it suitable for modeling and effectively identifying the inherent randomness and stochasticity in EEG signals^41^.

**Figure 4:**
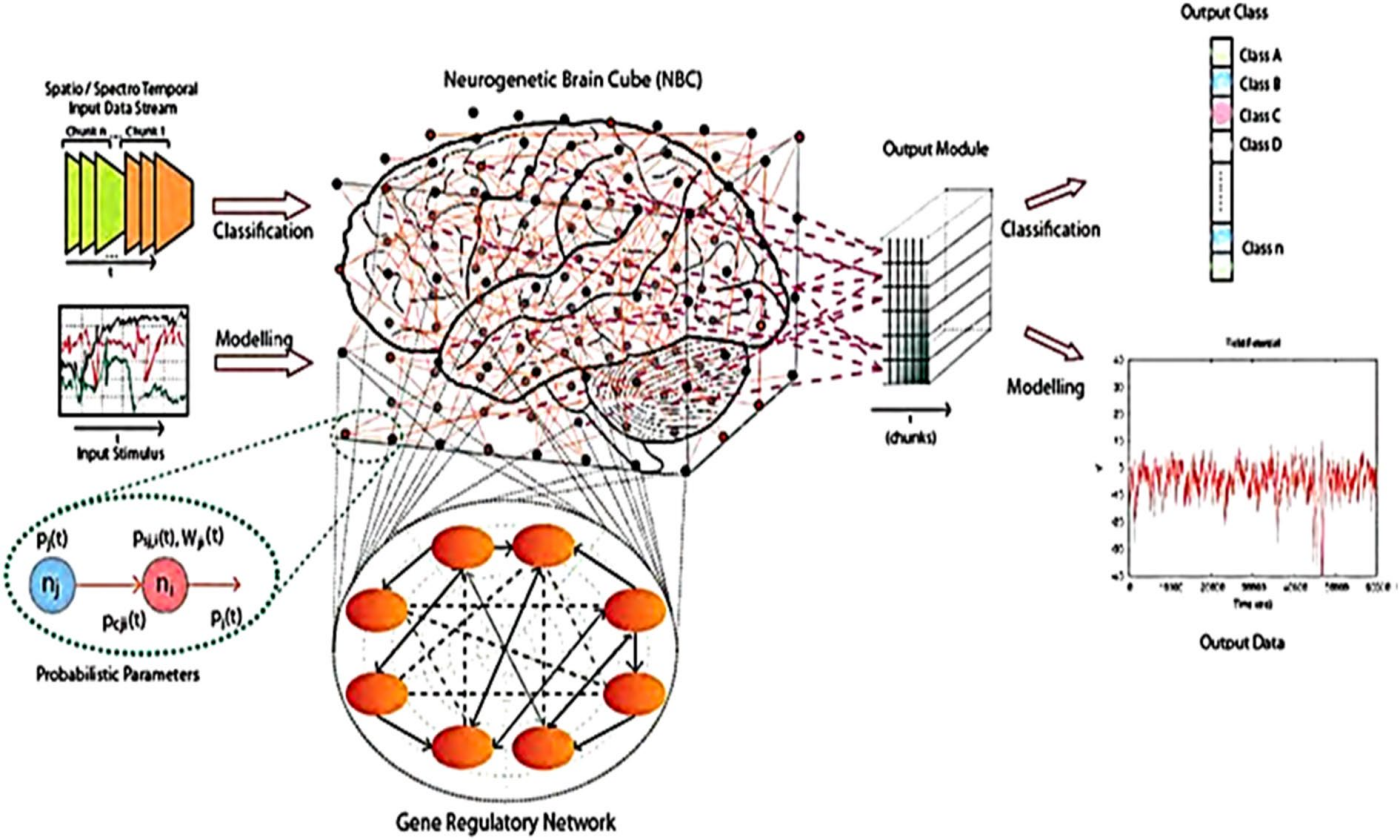
The NeuCube architecture (from^16^).

The NeuCube SNN architecture^16^ is shown in Fig. 4. It is constructed of three main modules, namely, a spike encoder, a 3D reservoir SNN, and an output classifier—a dynamic evolving SNN (deSNN)^42^.

The 3D SNN reservoir (SNNcube) is structured according to a brain template (e.g. Talairach, MNI, DTI) so that each spiking neuron represents a spatial area of this template. Potentially, gene regulatory networks can be included as well. In this way the SNNcube can learn and capture in its connections spatio-temporal associations from incoming input time series, such as EEG. That leads to a better accuracy and a better explainability of the model^33^.

In the upcoming section, we explore the outcomes of EEG data classification and scrutinize the activity patterns of neurons in each of the three models. This helps to understand the brain processes at operation more thoroughly and uncover the mechanisms underlying the discoveries. We can get important insights into the neural activity of the three networks, deep BiLSTM, RSM-SNN, and NeuCube, and their potential to deliver further knowledge about the effects of illnesses on the brain.

### Data specification

EEG data is recorded from 11 channels, including Fp1, Fp2, C3, C4, O1, O2, F7, F8, T3, T6, and Cz. The sampling frequency is set at 124 Hz, with a signal length of 500 samples, and a mini-batch size of 25. The EEG data is collected using the Natus machine 32-channel EEG based on the 10–20 system montage. Data were collected from the Long-term EEG Monitoring Center of the University Hospital (Imam Khomeini). The research was approved by the Research Ethics Committee of the Neuroscience Research Institute of the Tehran University of Medical Sciences. In this study, data pre-processing involved a bandpass filter (0.5–70 Hz) and a notch filter (50 Hz) for noise rejection. These steps aimed to enhance signal quality by isolating relevant frequency components and removing power line interference. Notably, no additional pre-processing beyond these filters was applied, ensuring transparency in data handling. These measures were crucial for optimizing the signal-to-noise ratio and isolating EEG-related features for subsequent analysis. EEG signals are categorized into three distinct groups within this study: 6 individuals diagnosed with epilepsy, 15 participants experiencing migraines without aura, and 15 healthy subjects. It is noteworthy that all data included in the analysis correspond to the interictal phase for both migraine and epilepsy. Specifically, the recorded data does not encompass any instances of attacks for either condition. Table 1 provides a comprehensive summary of the recorded EEG data, offering essential information for further analysis and interpretation.

**Table 1:** EEG dataset specifications.

Subjects	Status	Gender	Age
1	Epilepsy	Male	9
2	Epilepsy	Female	17
3	Epilepsy	Male	12
4	Epilepsy	Female	10
5	Epilepsy	Male	22
6	Epilepsy	Female	14
7	Migraine	Male	46
8	Migraine	Female	37
9	Migraine	Female	31
10	Migraine	Female	21
11	Migraine	Male	24
12	Migraine	Female	48
13	Migraine	Male	57
14	Migraine	Female	22
15	Migraine	Male	26
16	Migraine	Female	14
17	Migraine	Male	31
18	Migraine	Female	14
19	Migraine	Female	27
20	Migraine	Female	54
21	Migraine	Female	22
22	Migraine	Female	45
23	Healthy	Male	40
24	Healthy	Female	43
25	Healthy	Female	53
26	Healthy	Male	43
27	Healthy	Female	23
28	Healthy	Male	37
29	Healthy	Male	52
30	Healthy	Male	25
31	Healthy	Female	23
32	Healthy	Female	26
33	Healthy	Male	23
34	Healthy	Female	17
35	Healthy	Female	52
36	Healthy	Male	17

**Figure 5:**
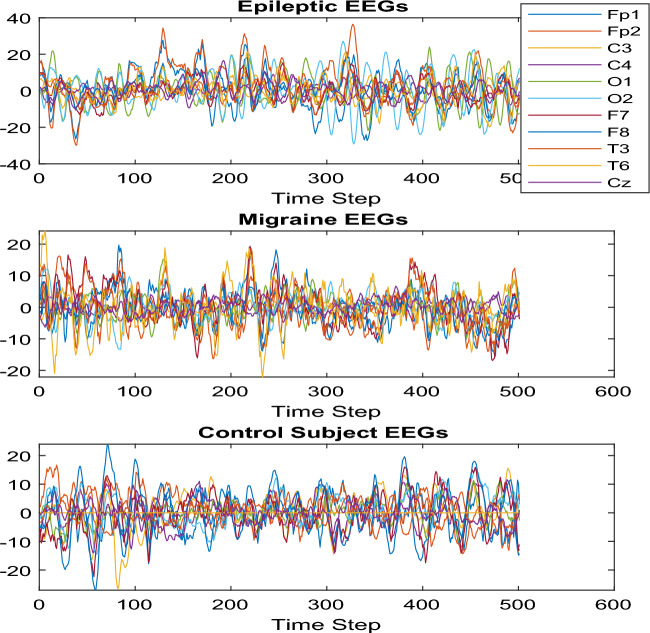
Three samples of EEG, including Fp1, Fp2, C3, C4, O1, O2, F7, F8, T3, T6, and Cz: (**a**) Epileptic data with high synchronizations in 11 channels; a generalized seizure is illustrated in this sample; (**b**) migraine EEG data samples; (**c**) normal EEG samples.

Figure 5 displays the EEG data samples for cases of Epilepsy, Migraine, and Normal condition. The recorded data are in open eyes status for the subjects. Epilepsy data are recorded from patients with focal epilepsy and refractory condition.

### Ethical approval

The data utilized in this study were previously collected by Prof. Tafakhori’s group under ethical protocols and made available for research. Informed consent was obtained from all subjects and/or their legal guardian(s) prior to the collection of the data. The use of this data was done in accordance with ethical guidelines and regulations that exist on issues related to informed consent, confidentiality, and other ethical considerations.

## Results

### Comparative analysis of the classification performance of BiLSTM, RMS-SNN and NeuCube on the case study data

This section presents new methods and results of our simulation and performance evaluation of three neural network structures: deep BiLSTM, RSM-SNN, and NeuCube. Simulations are conducted using MATLAB software, and the data are received from Imam Khomeini Hospital in Tehran, as discussed in the following sections. The source code for our proposed algorithm is available on GitHub at the following link: https://github.com/SSaeedinia/EEG-Classification-_Epilepsy-vs-Migraine. Additionally, for the classification mode of operation, we employed the NeuCube software, which can be accessed and run from http://www.kedri.aut.ac.nz/neucube.

Our goal in this section is to evaluate the behavior of three deep-learning neural networks. Additionally, we aim to investigate whether we can establish a logical connection between the patterns of neurons and the biological concept of the effect of migraine and epilepsy disorders on the brain’s neural activities. To achieve these objectives, we first evaluate the differences in the recorded signals in all three groups. We then assess the classification accuracy results and the effect of the number of neurons in the three mentioned models. By evaluating the activities of the neurons, we seek to establish a logical connection between the nature of the mentioned neurological disorders and the behavior of the networks, such as informative patterns and diagnostic biomarkers.

**Figure 6:**
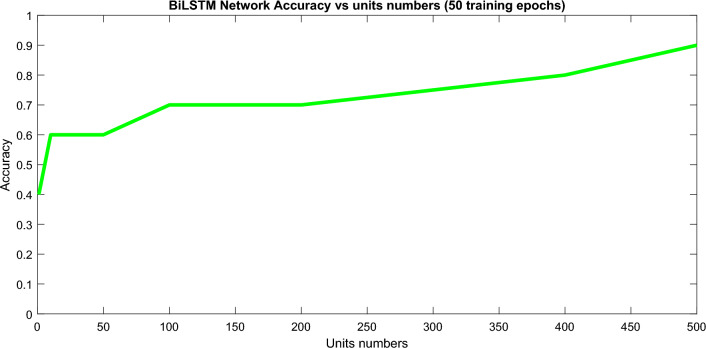
Dependency of the presented BiLSTM network classification accuracy on hidden units’ numbers.

**Figure 7:**
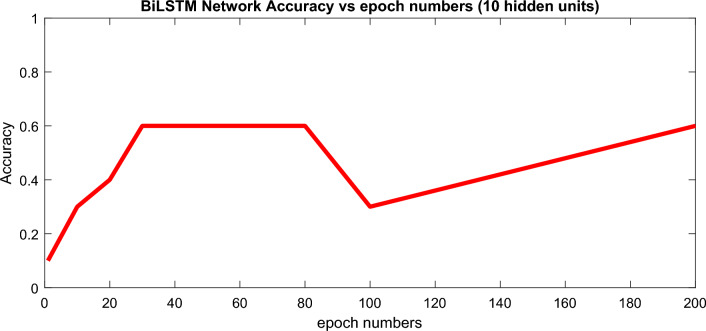
Evaluating epoch number’s effects on the classification accuracy.

All models were trained and tested in the leave-one-out cross-validation method. Figure 6 presents the results of the data classification accuracy of the deep BiLSTM structure based on hidden-unit numbers with 50 training epochs. A learning factor of 0.001 was used in our experiments. Based on the results presented in Figs. 6 and 7, it can be observed that higher hidden units require fewer training epochs to achieve optimal performance. However, Fig. 7 also indicates that increasing the number of training epochs does not necessarily lead to better results, and overtraining should be considered in deep learning networks. Therefore, it is important to determine an optimum value for the maximum number of training epochs. Figures 6 and 7 present the results of our data classification accuracy of the BiLSTM structure based on hidden-unit numbers with 50 training epochs.

As can be seen in Fig. 6, that the classification accuracy of the dataset directly depends on increasing the number of hidden units. To more delve into the details, Fig. 7 investigates the accuracy level dependency on the number of training epochs in BiLSTM with 10 hidden units.

A learning factor of 0.001 was used in our experiments. Based on the results presented in Figs. 6 and 7, it can be observed that higher hidden units require fewer training epochs to achieve optimal performance. However, Fig. 7 also indicates that increasing the number of training epochs does not necessarily lead to better results, and overtraining should be considered in deep learning networks. Therefore, it is important to determine an optimum value for the maximum number of training epochs.

**Figure 8:**
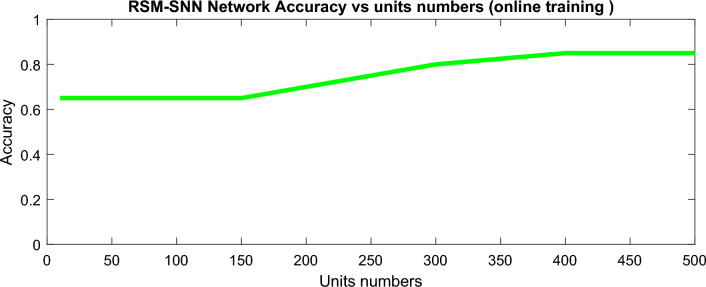
Dependency of the presented RSM-SNN network classification accuracy on hidden units’ numbers.

In Fig. 8, the accuracy of classification achieved by the Reservoir Spiking Neural Network (RSM-SNN) is visually presented. The classification accuracy is showcased through the utilization of a classifier that relies on the firing rate threshold of the features’ spike output generated by the observed neurons. This is a critical demonstration of the RSM-SNN’s robust classification capabilities, emphasizing on its effectiveness in accurately categorizing input data.

Moreover, Fig. 8 delves into the examination of optimal reservoir size in our study. The analysis systematically explores the impact of increasing reservoir size on classification accuracy. The findings unequivocally highlight a crucial threshold: for reservoirs surpassing 400 neurons, a discernible occurrence of overfitting becomes evident. This crucial insight informs the design considerations for optimizing the reservoir size in RSM-SNN models, ensuring a delicate balance between model complexity and overfitting avoidance.

The RSM-SNN has a lower accuracy rate of 0.85 when compared to BiLSTM’s accuracy rate of 0.9. However, the proposed RSM-SNN has a lower dependency on hidden neurons than the deep BiLSTM as well as lower computation time is required for SNN, conducted in the same processing device. SNN requires almost 5 min for the processing by 300 neurons while BiLSTM requires 27 min. processor is Duo cpu 2.13 GHz.

**Figure 9:**
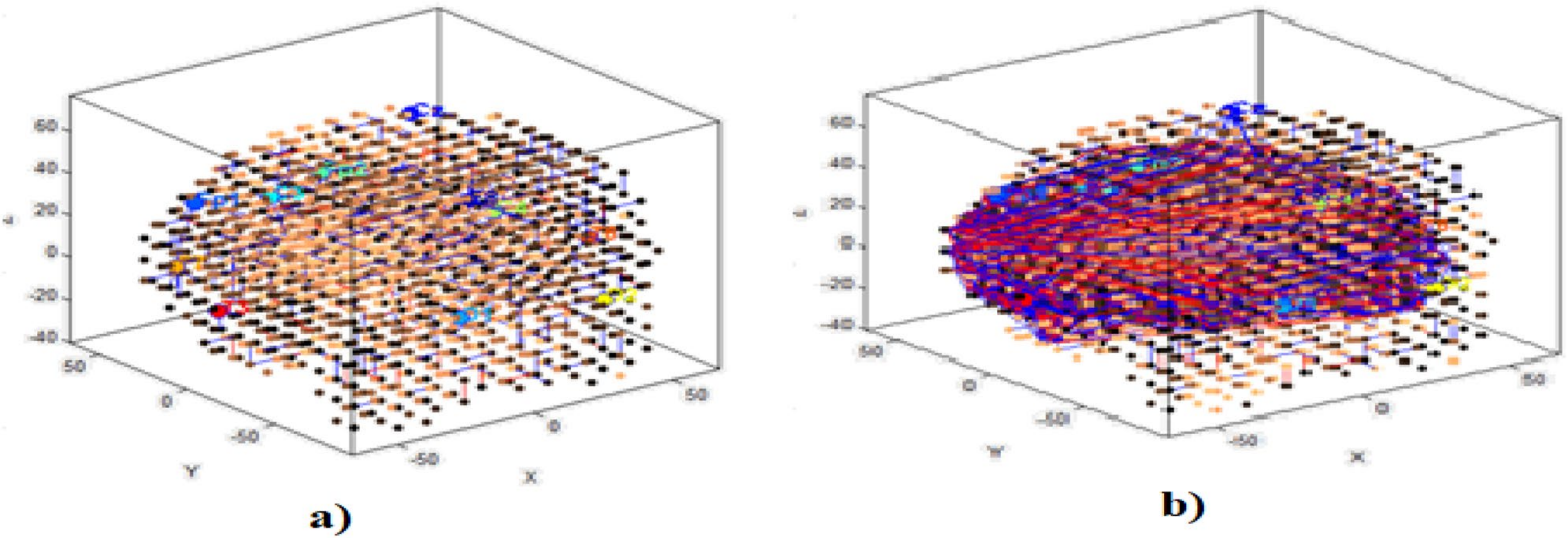
(**a**) Initial 3D SNN cube, mapping a Talairach template and the 11 EEG channels based on the 10–20 system; (**b**) the connectivity of a trained SNNcube on the three classes data.

Before a NeuCube model was trained, the 3D SNN reservoir was initialized with the Talairach brain template, so that every neuron in the 3D SNN cube corresponds to an area of the template. Figure 9a presents the 10–20 map-ping of the 11 EEG channels in the initialized NeuCube SNN reservoir. Figure 9b presents the connectivity of a trained SNN cube on the three classes of data. If only temporal data is used without spatial locations of the input variables, other methods can be used to map the variables into the 3D SNN structure while still preserving the temporal similarity between the temporal variables^28^.

The following parameter values are used in the NeuCube model: spike encoding threshold 0.5; STDP learning rate 0.01; Firing threshold 0.5; Refractory period 6; Modulation factor (for the deSNN classifier^42^) 0.8; Drift (for the deSNN) 0.005; kNN (for the deSNN) 3; number of training iterations 1^6,25^.

**Figure 10:**
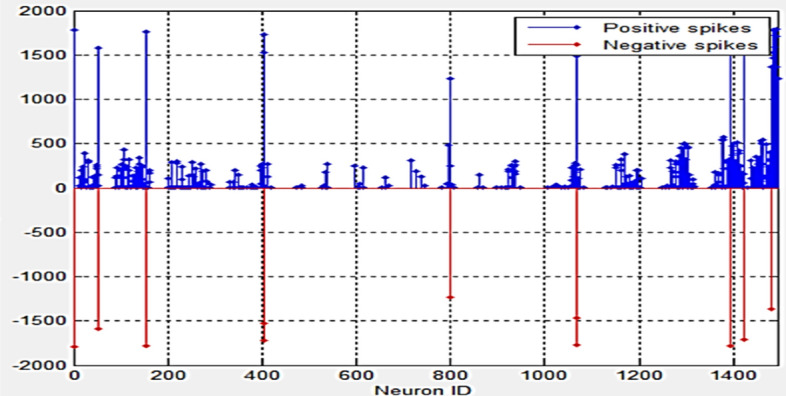
The spiking activity of the SNN cube during training.

Figure 10 shows the spiking activity of the SNN cube during training. The negative spikes are generated by the input neurons as a result of the spike encoding algorithm.

For classification purposes, the NeuCube architecture uses a dynamic evolving SNN classifier (deSNN)^42^. The classification test accuracy of a NeuCube model in a leave-one-out method to discriminate the three classes was 97%. When used to discriminate only the classes of epileptics from migraine data, the accuracy was perfect. This is the best result when compared with the other two methods.

### The proposed method for the discovery of patterns in the BiLSTM hidden neurons

**Figure 11:**
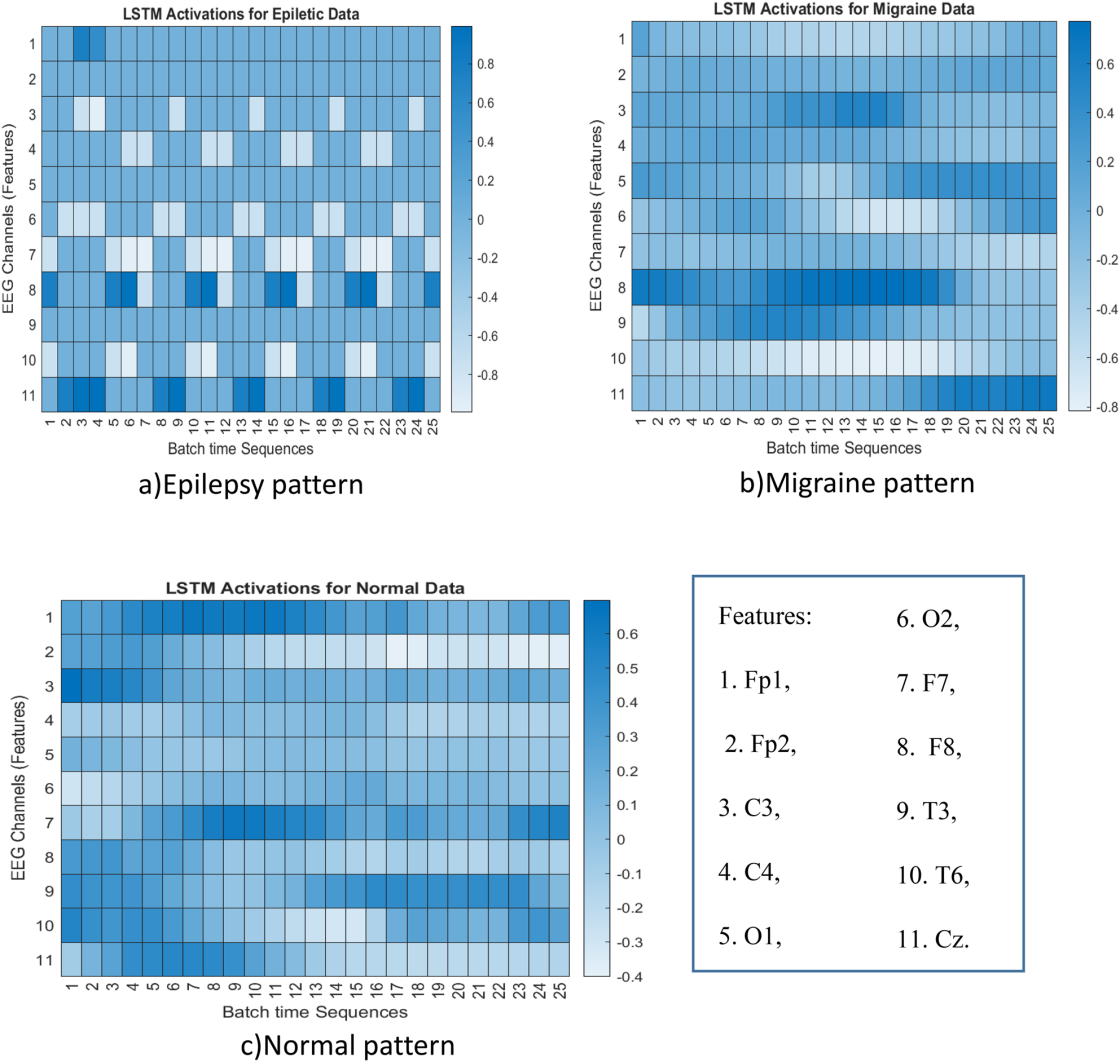
Activation patterns of BiLSTM for input features; here are EEG channel batch sequences for (**a**) Epileptic data. (**b**) Migraine EEG data and (**c**) normal condition.

This section investigates the activity of the deep BiLSTM network in three areas: the weights between the input and hidden layers, the weights between the hidden layers and output, and the characteristics of the input and batch data sequences. Figure 11 presents an evaluation of the state of the network weights. It illustrates that the patterns generated by the BiLSTM model exhibit a logical relationship between the inherent abnormalities. Specifically, Fig. 11-a shows a pattern of oscillations that is consistent with the effects of seizures on neural activity. During the ictal phase and even in the interictal phase, higher synchronization and sharp oscillations can be observed in EEG patterns. Therefore, the activation patterns produced by the model can provide insights into how epilepsy disorder affects EEG signals and brain neural activity.

Figure 11-b depicts the activity of the BiLSTM model and reveals an asymmetry between high and low activity in some channels, particularly in the occipital lobe. This finding is consistent with the inherent effects of migraine disorder on the brain, although the asymmetry is not as clear as in the abnormal patterns shown in Fig. 11-a. In contrast, Fig. 11-c shows a normal condition without any asymmetry or oscillation, which is not indicative of any abnormality. Therefore, the activity patterns generated by the BiLSTM model exhibit biologically plausible patterns that are consistent with the effects of abnormalities.

**Figure 12:**
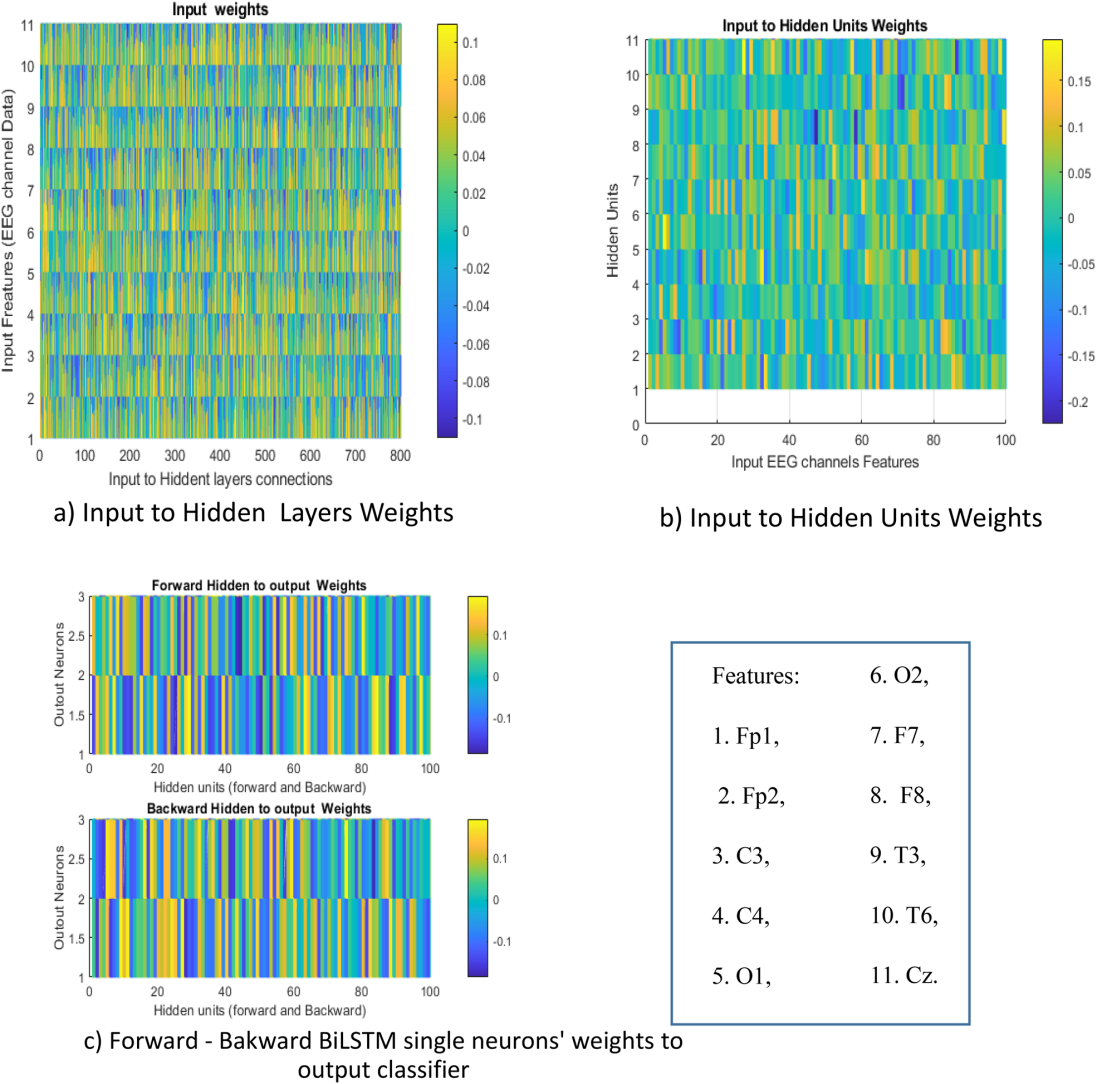
Hidden weights connections in BiLSTM (**a**) the weights connections from input to hidden neurons of BiLSTM forward–backward and 3 fully connected layers with 100 hidden units in BiLSTM, (**b**) product of input to hidden units weights, (**c**)forward–backward BiLSTM single neurons connection weights to the output layer.

The connections between the layers of the networks, particularly in Fig. 12-a, do not provide significant criteria for distinguishing differences based on the features. However, in Fig. 12-b, we can observe that the weight connections between hidden units and input layer, including data channels 8, 9, and 10, which correspond to the channels F8, T3, and T6 in EEG, are more amplified in a negative direction in some units’ connections (indicated by dark blue rectangles). Figure 12-c also indicates that the activation degree of output node 1 is almost higher than 2, and the activation degree of node 2 is greater than 1. This finding suggests that since higher synchronization is one of the important features of an epileptic network, it is logical that the connections of the classifier are trained to manifest a higher activation degree for the classification node. However, the weight connections cannot distinguish a meaningful link between disorder effects. Clearly, further research is needed to better understand the connections between the layers of the networks and their relationship to disorder effects.

### RSM-SNN observed neurons firing patterns

**Figure 13:**
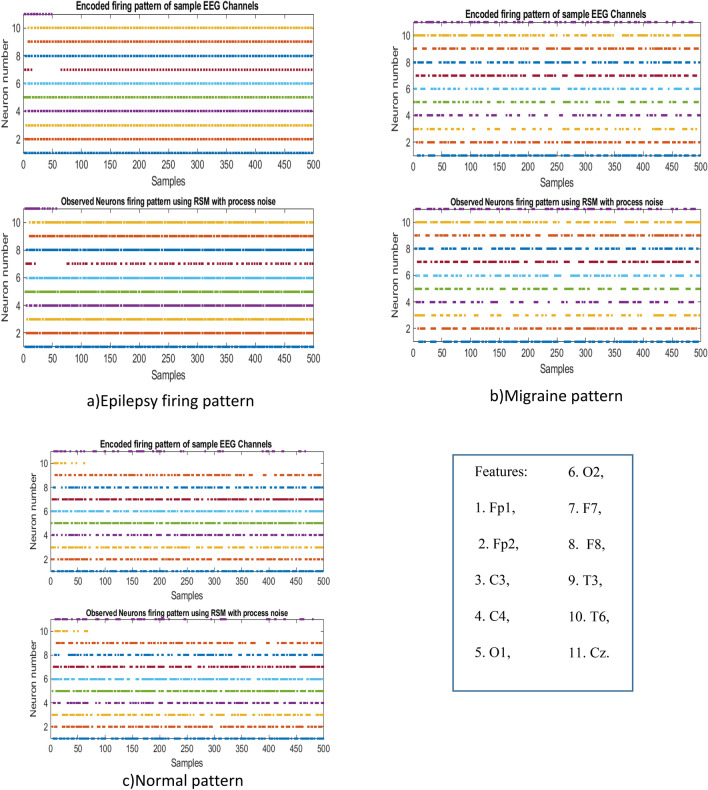
Output firing rate of observed neurons under the proposed online spike detection algorithm with input data of the classes (**a**) Epilepsy (**b**) migraine and (**c**) normal condition patterns.

The specifications and simulation parameters of the RSM-SNN are provided in Table 2, while the firing patterns of observed and hidden neurons are shown in Fig. 13. To classify the output, the RSM-SNN uses a simple threshold-based approach, where the firing rate threshold is determined by training the network on 70% of the dataset. This approach is integrated into the RSM-SNN’s output classifier, which helps to accurately classify the input data.

**Table 2:** RSM-SNN simulation parameters.

Parameter	Description	Value
*dt*	Time discretization interval	1 ms
$\tau$	Membrane potential time constant	40
$\vartheta$	Firing threshold	0
$\Delta v$	Firing sensitivity to the membrane potential	10
a, b, c and d	Izhikevich neuron parameters	0.06, 0.45, − 50 and 2
Observed Neurons	Izhikevich neurons in RS-SNN receive encoded data, and their firing pattern is considered as the output, learning law is ReSuMe	11
Hidden Neurons	Izhikevich neurons which do not receive any external input and their weights are updated based on STDP law	289

Based on the data presented in Fig. 13-a, b, and c, it is clear that feature inputs 7 and 10, specifically the F7 and T6 EEG channels, are crucial in distinguishing between epilepsy, migraine, and normal conditions. These findings are consistent with the results obtained from BiLSTM hidden activity, but the firing pattern of RSM-SNN neurons offers even more precise information.

**Figure 14:**
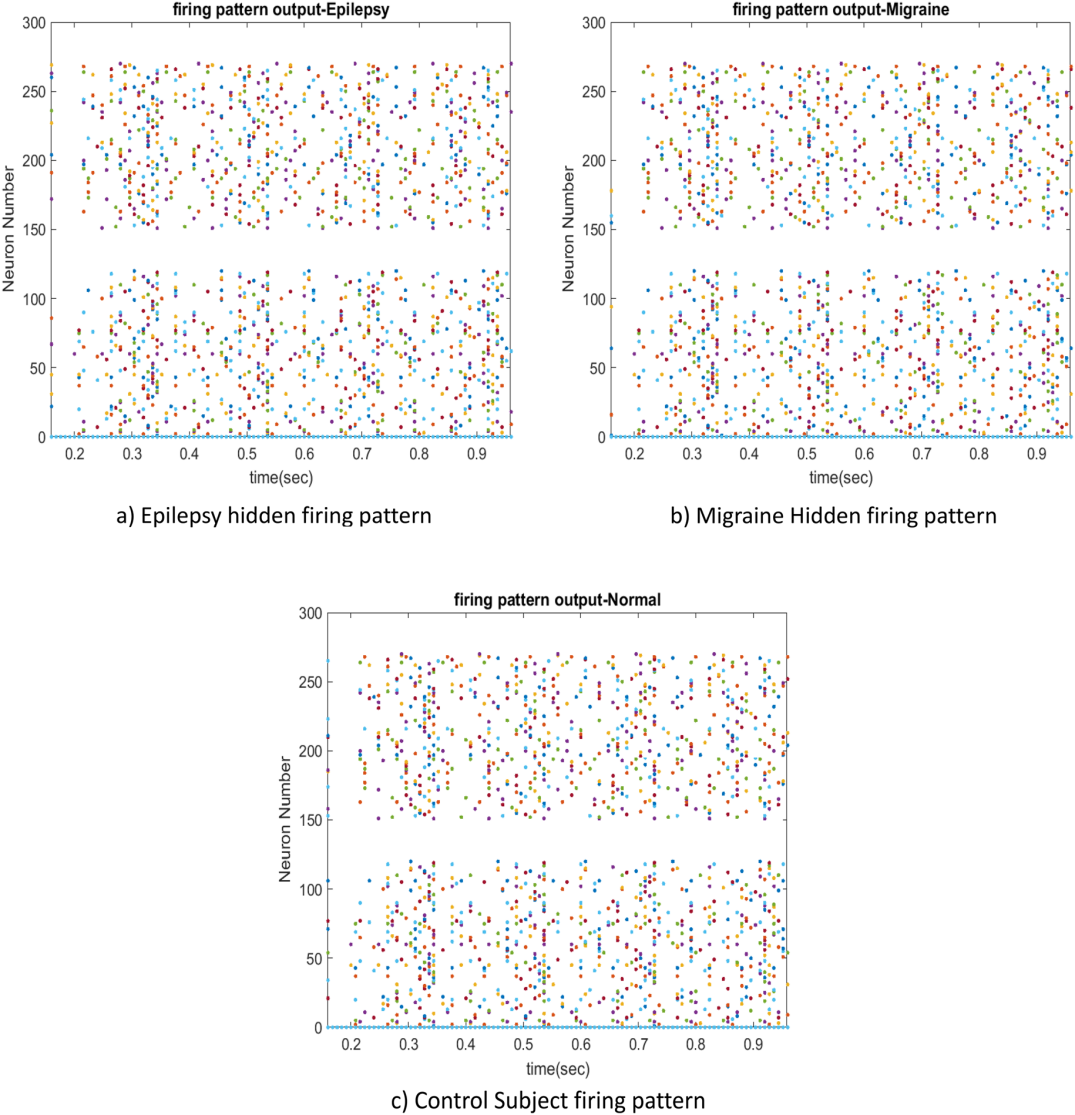
Hidden units firing patterns of 289 hidden neurons, (**a**) Epilepsy (**b**) Migraine, and (**c**) normal firing patterns.

While the observed neurons can provide classification criteria based on firing rate, Fig. 14 reveals that the hidden units are unable to provide specific and significant distinguishable criteria for classifying data into the three aforementioned classes within the proposed deep RSM-SNN.

### Discovering EEG diagnostic biomarkers from the NeuCube models

NeuCube models are trained on the case study data, the first one is trained on 3 classes of data (epilepsy, migraine, and normal), and the second one, on 2 classes of data—epilepsy and migraine. The purpose was to try to establish diagnostic biomarkers for the 3 and also for the 2 classes of brain states.

In both cases, the method consists of the following steps:

- Train a NeuCube model for classification, so that the 3D SNN cube is trained with the use of STDP unsupervised learning and after that, the deSNN classifier is trained;
- Extract a feature interaction network (FIN) graph from the trained 3D SNNcube, so that the arcs represent the number of spikes exchanged between the two neurons during training representing also time association between every two neurons, i.e. if one input neuron spikes at a time *t* the other one spikes in the next time moment (t + 1). This indicates a temporal association between the two EEG channels represented by the two input neurons.
- The most active neurons in terms of spike generation and spike exchange can be considered as biomarkers for the discrimination of the data categories.

**Figure 15:**
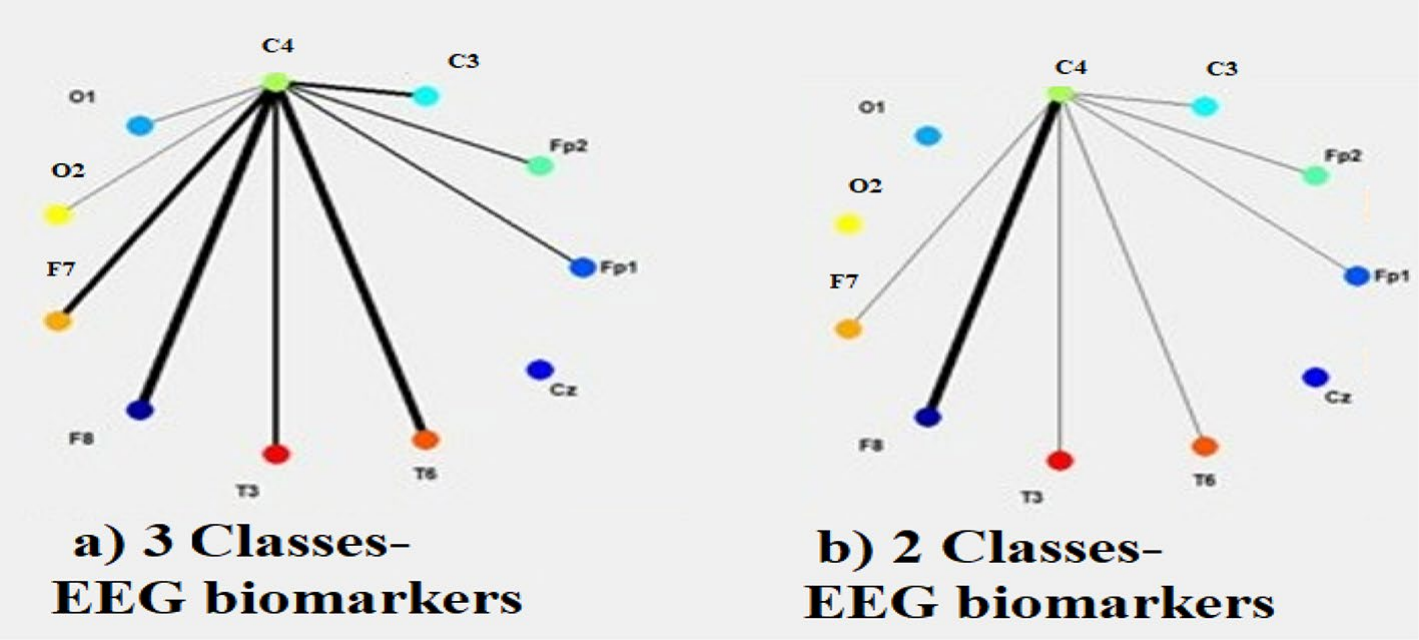
(**a**) Feature interaction network (FIN) graphs for the NeuCube model trained on all three classes data; (**b**) FIN of a NeuCube model trained only on epilepsy and migraine data.

Figure 15 represents the level of information exchange activity between input features (EEG channels) of the NeuCube-based models. Stronger activities determine EEG biomarkers.

Figure 15 suggests that when classifying two exclusive classes of the human brain, specifically migraine and epilepsy states, C4 and F8 exhibit efficacy as biomarkers. However, when distinguishing between three classes, namely Migraine, epilepsy, and control subjects, the probability of overlapping between migraine and control subjects may increase. Therefore, an additional EEG channels, namely T6 and F7, must be added to the set of biomarkers.

### Evaluation of the performance of the proposed spike encoding algorithm

**Figure 16:**
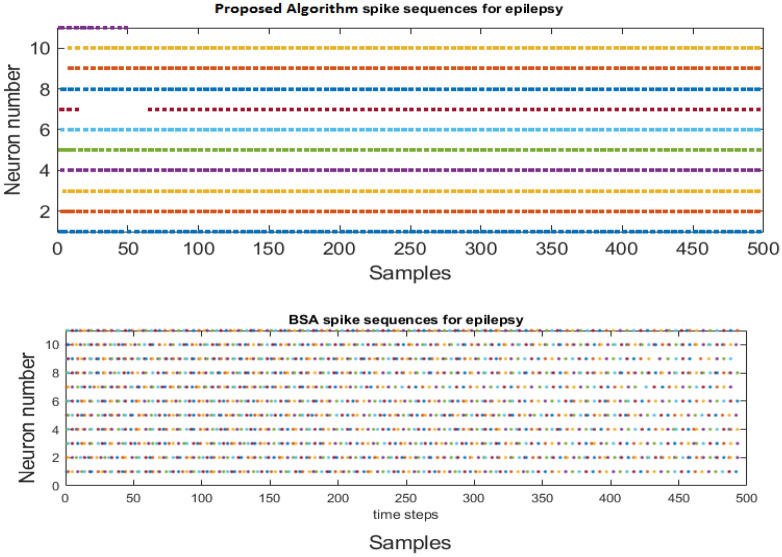
Comparison of our proposed encoding algorithm with the popular BSA algorithm.

In this section, we compare the traditional BSA encoding algorithm (see ^33,37^) with the proposed in this paper one. As depicted in Fig. 16, both encoding approaches generate a high frequency of spiking patterns, corresponding to the epileptic disorder effects on neural activities, but our proposed algorithm is able to detect more intricate details.

**Figure 17:**
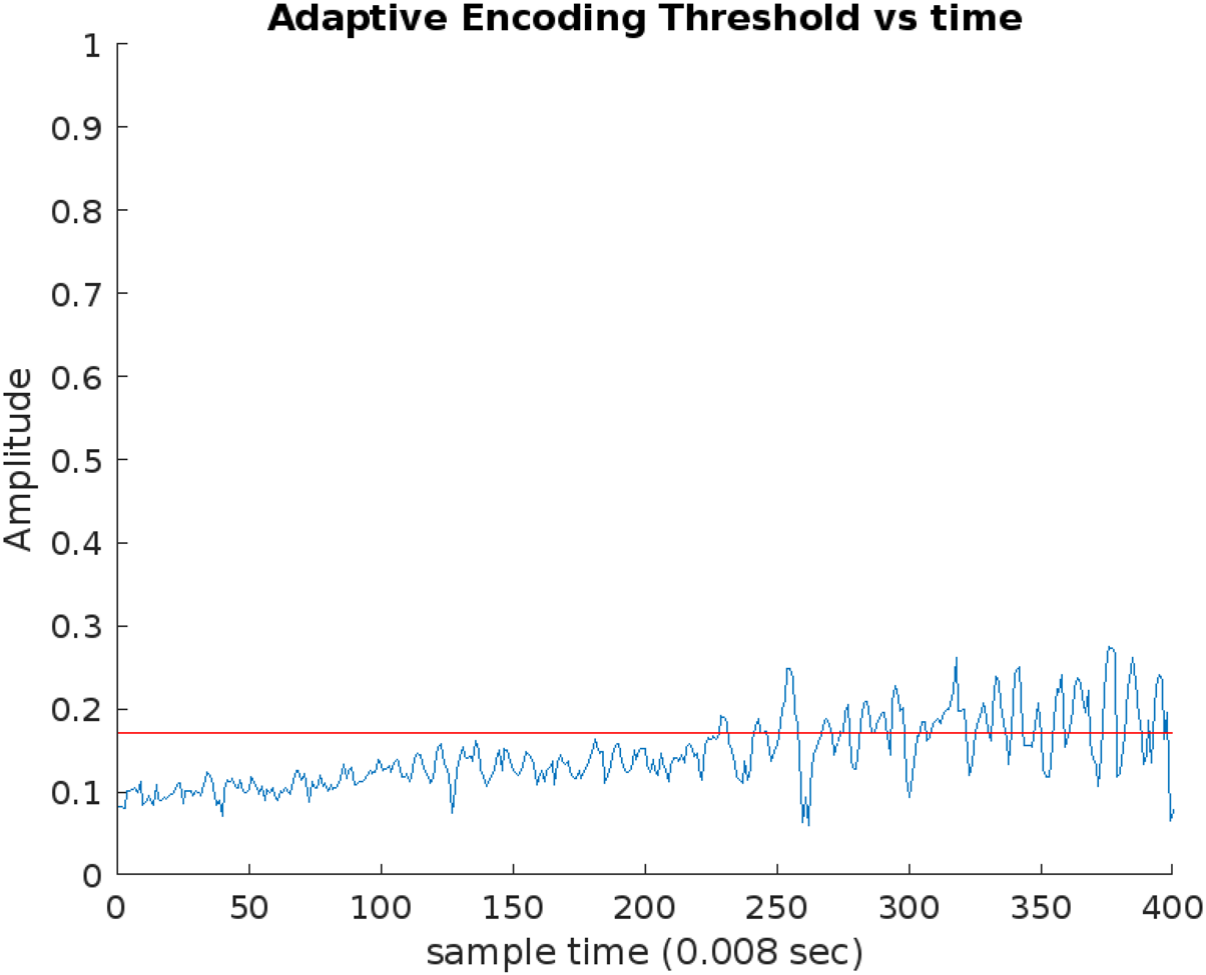
Adaptive encoding threshold calculated by the proposed spike encoding algorithm versus time samples.

To understand the dynamic adaptation of the threshold in our proposed algorithm, Fig. 17 illustrates the process of threshold adaptation over time samples in the encoding mechanism. The figure provides a visual representation of how the calculated threshold undergoes an oscillatory convergence, gradually settling toward a specific and stable threshold value. This visualization enables a clear observation of the threshold adaptation dynamics, highlighting the oscillatory nature of the process and emphasizing the ultimate convergence to a well-defined threshold.

Figure 17 provides insightful information on the online normalized EEG signals within our dataset. Notably, it unveils that the mean threshold across all 11 channels signals is approximately 0.18.

### Classification performance comparison across machine learning methods

In this section, a thorough comparison is conducted among the three methods utilizing 1000 hidden units, ensuring a fair evaluation of our dataset. The results are succinctly presented in Table 3, showcasing the accuracy and processing time metrics for RSM_SNN, BiLSTM, and NeuCube on our specific dataset. This comprehensive analysis provides valuable insights into the performance characteristics of each method, allowing for an informed assessment of their effectiveness in handling the given data.

**Table 3:** A classification performance comparison of the three proposed for EEG data analysis models, RSM_SNN, BiLSTM, and NeuCube versus other popular machine learning methods demonstrate the advantage of the studied models.

Methods	Accuracy (%)	Execution time for one test sample (sec)
RSM_SNN	85	1.8
BiLSTM	80	3.92
NeuCube	97	3.2
SVM (Entropy feature)	60	0.083
Random Forest (Entropy feature)	65	0.071

Table 3 presents notable accomplishments in EEG classification. The NeuCube framework demonstrates the highest accuracy in identifying epilepsy versus migraine and normal EEGs within the dataset, indicating its effectiveness in approximating spatial brain areas based on EEG data acquisition atlases. This high accuracy can be justified by the fact that NeuCube integrates spatial and temporal features, unlike the other approaches that only utilize temporal features. Additionally, NeuCube is a type of reservoir structure that benefits from the advantages of rSNN and utilizes the LIF neuron model as a biologically plausible neuron model. Therefore, it is reasonable to expect that NeuCube has the potential to surpass the rSNN model due to its consideration of more features, including spatial information, which is essential in EEG classification. The justification for the perfect accuracy of NeuCube in classifying epilepsy and migraine EEGs lies in its ability to integrate spatial and temporal features, unlike other approaches that only utilize temporal features. Additionally, NeuCube’s utilization of the LIF neuron model as a biologically plausible neuron model and its reservoir structure, which benefits from the advantages of rSNN, contribute to its potential to achieve high accuracy in EEG classification.

### Advantages and limitations of the proposed models

The use of spiking neural networks (SNNs) for the classification of EEG signals associated with neurological ailments has shown promise. Numerous investigations have illustrated that SNNs are effective in energy use and expeditious processing, rendering them a fitting technique for the classification of EEG signals in applications that require real-time inspection^12,34^. SNN-based techniques for EEG classification often lag behind deep neural network techniques in terms of performance, but DNNs require substantial datasets to attain their optimal performance, a circumstance that is not always feasible with smaller datasets^43^. Thus, it is imperative to meticulously contemplate the training algorithm and feature extraction approach when employing SNNs to establish the most advantageous course of action, particularly within the confines of limited training datasets or online processes involving wearable devices.

In our comparative study, the proposed SNN architecture, which solely encompasses temporal features, was compared with the NeuCube and BiLSTM methodologies for the classification of EEG signals linked to epilepsy and migraine. It is noteworthy, that this paper assumes that the patient is suspected of having epilepsy or migraine without concurrent any other brain disorders.

While all three methodologies generated satisfactory results, the proposed RSNN exhibited a shorter processing time than NeuCube and BiLSTM. The proposed here spike encoding algorithm for the RSNN necessitates convergence toward the optimal encoding threshold. Nevertheless, the speed of convergence is commendable, as evidenced by our dataset. Furthermore, it is worth noting that the proposed RSNN failed to surpass NeuCube in terms of classification accuracy. Therefore, when selecting a suitable classification method for EEG signals, it is essential to consider the specific demands of the application and the trade-offs between speed and accuracy, particularly within the context of neurological disorders.

## Discussion and conclusion

This paper introduces methods for the creation of an online intelligent diagnostic systems, aimed at distinguishing between epilepsy, migraine, and normal EEG signals. Three key concerns are addressed: the effectiveness of SNN-based and ANN-based approaches on small datasets; the utilization of the novel RSM-SNN structure for pattern recognition in biological signals, and a comparison between BiLSTM, NeuCube, and the proposed RSM-SNN architectures for EEG classification.

The study’s focus on the RSM-SNN classifier showcases its superiority over BiLSTM in terms of accuracy and processing time. Results reveal an 85% accuracy for RSM-SNN, outperforming BiLSTM’s 80% when both structures are composed of 1000 hidden units. Importantly, the proposed RSM-SNN method exhibits a remarkable 54% reduction in processing time compared to BiLSTM, making it highly applicable for real-time applications. NeuCube, leveraging spatiotemporal features, achieves 97% accuracy but with almost 49% longer processing time compared to the proposed RSM_SNN.

Additionally, the study explores the impact of EEG channels on classification. BiLSTM identifies F8, T3, and T6 as crucial, while RSM-SNN highlights F7 and T6. NeuCube, the best-performing model, suggests C4, F8, T6, and F7 as discriminative channels. Despite differences in learning approaches, all models converge on a subset of common features, emphasizing on their significance.

The proposed models demonstrate a high classifier’s efficacy in EEG classification, offering a compelling compromise between speed and accuracy. The identified EEG channels across the models, especially F8 and C4, contribute to the understanding of epilepsy and migraine disorders.

It should be noted that different studies have identified different EEG channels as important for the problem at hand. For instance, a study^37^ proposed that T3, F7, O1, and O2 channels are the most decisive for the diagnosis of migraine, based on PSD magnitude increase under flash stimulation.

While acknowledging the small dataset limitation, the biomarker discoveries in our study provide a foundation for future investigations. The proposed methods are deemed valid for learning and feature discovery, encouraging further testing on larger datasets and clinical applications.

While the paper suggests new diagnostic biomarkers to discriminate between epilepsy, migraine, and control, further studies can be directed toward their predictive abilities. A new study demonstrates that a NeuCube model can be used as a spatio-temporal associative memory so that once trained on full-scale time data, it can predict outcomes based on only partially presented input data in time and also using a smaller number of channels^44^. Early prediction of the onset of epilepsy or migraine is an important task for future research.

In conclusion, this paper contributes valuable insights into deep learning neural networks for classification, emphasizing on the importance of their speed, accuracy and explainability. The development of SNN models holds promise for implementing implantable diagnostic devices, advancing early diagnosis in an online mode.

### Supplementary Information

Supplementary Information.

## Data Availability

All data generated or analyzed during this study are included in this published article and its supplementary information files.
